# Incidence and risk factors of retinopathy of prematurity in three neonatal intensive care units in Palestine

**DOI:** 10.1186/s12886-019-1180-4

**Published:** 2019-08-20

**Authors:** Mohammad T. Akkawi, Mohammad M. Shehadeh, Amjaad N. Abu Shams, Doaa M. Al-Hardan, Lara J. Omar, Omar H. Almahmoud, Jamal A. S. Qaddumi

**Affiliations:** 1 Department of Special Surgeries, Faculty of Medicine and Health Sciences, An-Najah National University Hospital, An-Najah National University, Nablus, Palestine; 2 Ophthalmology Department, An-Najah National University Hospital, Nablus, West Bank, Palestine; 3 An-Najah National University Hospital, Nablus, Palestine; 4 Instructor - Pharmacy, Nursing and Health Professions College, Birzeit University, Birzeit, Palestine; 5 Faculty of Medicine and Health Sciences, An-Najah National University, PO box 7, Nablus, Palestine

**Keywords:** Incidence, Palestine, Retinopathy of prematurity, Risk factors

## Abstract

**Background:**

Severe Retinopathy of Prematurity (ROP) is a serious vasoproliferative disorder that can affect extremely premature infants. It continues to be one of the most important preventable causes of blindness in children. Our study is aimed at finding the incidence of ROP and its association with some risk factors in Palestine.

**Methods:**

From the 1st of January 2016 to 31st December 2016, a total number of 115 infants who met the criteria for ROP screening in three neonatal intensive care units were included in the study. The medical records of infants were reviewed retrospectively and multiple factors that may be associated with the development of ROP were collected manually.

**Results:**

The incidence of ROP and severe type 1 ROP that require treatment was 23.5 and 11.3% respectively. After conducting univariate analysis of risk factors, statistically significant risk factors affecting the development of ROP in our study were: low gestational age, low birth weight, type of multiple gestation, the presence of affected sibling, low level of Hemoglobin at birth, respiratory distress syndrome, low Hemoglobin level, blood transfusion and days on oxygen supplements with either mechanical, non-mechanical methods or both combined. High bilirubin levels were found as a protective factor against the development of ROP. However, when a multivariate analysis was performed, only low gestational age, total days on oxygen supplement and high bilirubin levels were significant regarding the development of ROP.

**Conclusion:**

The incidence of ROP is considered a relatively low percentage compared to neighboring countries that have higher levels of human development index. Statistically significant risk factors need to be considered when clinicians deal with premature infants.

## Background

It’s estimated that the number of blind children in the world is approximately 1.5 million [1]. Among those cases, Retinopathy of Prematurity (ROP) is one of the most important preventable causes. Nearly 50,000 infants each year become blind from ROP worldwide [2].

Severe ROP is a serious vasoproliferative disorder that can affect extremely premature infants. ROP, also known as retrolental fibroplasia, was first recognized as a disease in 1942 by Dr. Theodore L. Terry [3]. Given the enormous impact of lifetime vision impairment or blindness caused by ROP on the quality of life of affected infants [4], many studies have been performed in order to find out the relationship between ROP and certain risk factors [5–8].

It was noticed that there is a variable risk of ROP among different countries in the world. For example, the incidence of ROP among low-to-medium human development countries was 47.2% in Nigeria [9] and 33.9% in India [10]. While in high-to-very high human development countries, the incidence was 26.1% in Iran [11] and 10.1% in Norway [12]. The purpose of our study is to find out the incidence and risk factors of ROP in Palestine as there is no such published data about ROP in Palestine.

## Methods

This study is a retrospective review approved by An-Najah National University’s Institutional Review Board (IRB) and has adhered to its rules and regulations. The study was based on information collected from patients’ files only. The study was performed in three neonatal intensive care units (NICUs) in North West Bank, Palestine: Specialized Arab Hospital, Nablus Specialized Hospital, and Arab Women’s Union Hospital. Eye examinations were performed on all infants with a birth weight of less than 1500 g (g) or with a gestational age of 32 weeks or less, as well as selected infants between 1500 g and 2000 g with an unstable clinical course who are believed to be at high risk by their attending pediatrician or neonatologist.

### Eye examination method

The initial examination was performed 4–6 weeks postnatally [13]. The exact timing of examination depends on when the NICU nursery call the retinal specialist and the availability of the doctor at that time. Topical tropicamide 0.5% and phenylephrine 2.5% were instilled every 15 min 4 times, one hour before examination. Topical anesthesia, eyelid speculum, indirect ophthalmoscope using 20 diopter lens, and scleral indentation were used in all examinations. Findings were classified according to the Revisited International Classification of Retinopathy of Prematurity [14]. If ROP was detected, the examinations were repeated at intervals according to the severity of the findings. Follow-up examinations were determined by the examining ophthalmologist on the basis of retinal findings classified according to the international classification [15].

### For purpose of data analysis, the infants were divided into 2 groups

- Group 1: Infants without ROP.
- Group 2: Infants with ROP.

The files of already screened infants from the 1st of January 2016 to 31st December 2016 were examined and multiple factors that may be associated with the development of ROP -according to the literature- were collected manually.

### Identification of risk factors

The assessed risk factors of ROP were categorized into demographic, neonatal characteristics, neonatal co morbidities detected and therapeutic interventions used.

- Demographic features: residency (West bank or Gaza) and gender.
- Neonatal characteristics: gestational age, birth weight, type of pregnancy (singleton or multiple gestations) and level of hemoglobin at birth.

Co-morbidities detected: sepsis, patent ductus arteriosus, intraventricular hemorrhage, respiratory distress syndrome, hydrocephalus, lowest hemoglobin and maximum bilirubin level before the date of first examination.

Therapeutic interventions: blood transfusion, surfactant use, mechanical ventilation and oxygen supplement.

### Definitions

The residency place of infants involved in the study was classified into two groups: West bank and Gaza, the two regions that make up the state of Palestine. Gestational age was ascertained from the maternal last menstrual period (LMP), or Ultrasound parameters if LMP was not certain. Sepsis was diagnosed based on positive blood cultures. Patent ductus arteriosus was confirmed by 2 dimensional echo. Serial transfontanelle ultrasound was used to diagnose intraventricular hemorrhage and hydrocephalus. Respiratory distress syndrome was diagnosed based on clinical and radiological characteristics.

### Statistical analysis

The statistical analysis was performed using the Statistical Package for Social Sciences program (SPSS) (IBM Corp. Released 2012. IBM SPSS Statistics for Windows, Version 21.0.Armonk, NY: IBM Corp.). Descriptive statistical methods were used to describe the study sample. Univariate comparisons of risk factors between the groups were evaluated using the chi square and independent T-test, with statistical significance taken to be *P* < 0.05. Fisher’s exact test was used when the expected cell count was less than 5. A multivariate comparison of statistically significant risk factor by hierarchical method of data entry was then preformed.

## Results

A total number of 115 infants who met the criteria for ROP screening in three NICUs were included in the retrospective study. ROP was diagnosed in 27 babies and severe type 1 ROP that require treatment [16] was diagnosed in 13 babies, the incidence of ROP and severe ROP was estimated to be 23.5 and 11.3% respectively.

### Demographic features

Regarding geographical distribution, 45 of the infants were from Gaza and 70 from the West Bank. ROP was diagnosed in 33.3% in Gaza vs. 17.1% in West Bank (*P =* 0.070). 68 infants were males, and 47 were females. Refer to Table 1.

**Table 1:** Demographic features of infants and their relation with ROP development

Variable	Category	No ROP % (*n* = 88)	ROP % (*n* = 27)	*P* value
Residency group	Gaza	30 (66.7%)	15 (33.3%)	0.070
	West bank	58 (82.9%)	12 (17.1%)	
Gender	Male	50 (73.5%)	18 (26.5%)	0.36
	Female	38 (80.1%)	9 (19.1%)	

% within the same variable group

*ROP* Retinopathy of Prematurity

### Neonatal characteristics

The average gestational age among those who were diagnosed with ROP was 29.4 ± 1.3 (mean ± standard deviation) weeks and 30.7 ± 1.4 weeks in the Non-ROP group (*P* < 0.0010). The average birth weight was 1185.0 ± 308.3 g among infants with ROP and 1382.5 ± 253.3 g in infants without ROP (P = 0.0012). Having a sibling –from the same gestation- affected by ROP was found to be of statistical significance regarding the development of ROP in those who were a part of multiple gestations (*P* < 0.0010). The average level of hemoglobin at birth was 15.7 ± 1.8 g/dl in the non-affected group and 14.9 ± 2.6 g/dl in the affected one (*P* = 0.043). Refer to Table 2.

**Table 2:** Neonatal characteristics of infants and their relation with ROP development

Variable	Category	No ROP % (*n* = 88)	ROP % (*n* = 27)	*P* value
Gestational age (weeks)	Mean ± SD	30.7 ± 1.4	29.4 ± 1.3	< 0.0010*
Gestational age group	≤ 30 weeks	29 (55.8%)	23 (44.2%)	< 0.0010*
	> 30	59 (93.7%)	4 (6.3%)	
Birth weight (grams)	Mean ± SD	1382.5 ± 253.3	1185.0 ± 308.3	0.0012*
Birth weight group	< 1000 g	4 (44.5%)	5 (55.5%)	0.0085*
	1000–1500 g	53 (74.6%)	18 (25.4%)	
	> 1500 g	31 (91.2%)	3 (8.8%)	
Type of pregnancy	Singleton	14 (73.7%)	5 (26.3%)	0.75
	Multiple gestation	74 (77.1%)	22 (22.9%)	
Multiple gestation	Twin	23 (82.1%)	5 (17.9%)	< 0.0010*
	Triplet	30 (85.7%)	5 (14.3%)	
	Quadruplet	21 (75%)	7 (25%)	
	Quintuplet	0	5 (100%)	
Sibling	Sibling affected by ROP	10 (37%)	17 (63%)	< 0.0010*
	Sibling not affected by ROP	61 (97%)	2 (3%)	
Level of HB at birth (g/dl)	Mean ± SD	15.7 ± 1.8	14.9 ± 2.6	0.043*

% within the same variable group

*significant at *p* value less than 0.05

*ROP* Retinopathy of Prematurity

*SD* Standard Deviation

*HB* hemoglobin

### Neonatal co-morbidities

Sepsis, patent ductus arteriosus, intraventricular hemorrhage and hydrocephalus were not statistically significant regarding the development of ROP (*P* = 0.84, 0.20, 0.37, 0.070 respectively). However, respiratory distress syndrome was significantly associated with ROP (*P* = 0.021). Among those without ROP, the lowest hemoglobin level before the date of first examination was found to have an average of 8.4 ± 1.4 g/dl, while in the affected group it was 7.8 ± 1.5 g/dl (*P* = 0.038). The average maximum bilirubin level before the date of first examination was found to be higher in the non-affected group compared with the affected one: 7.3 ± 2.4 mg/dl Vs. 5.0 ± 2.7 mg/dl (*P* < 0.001). Refer to Table 3.

**Table 3:** Neonatal co-morbidities

Variable	No ROP % (*n* = 88)	ROP % (*n* = 27)	*P* value
Sepsis	4 (4.5%)	2 (7.4%)	0.84
Patent ductus arteriosus	1 (1.1%)	2 (7.4%)	0.20
Intraventricular hemorrhage	3 (3.4%)	2 (7.4%)	0.37
Respiratory distress syndrome	57 (64.8%)	22 (81.5%)	0.021*
Hydrocephalus	0	1 (3.7%)	0.070
Lowest Hemoglobin level (g/dl)	8.4 ± 1.4	7.8 ± 1.5	0.038*
Maximum bilirubin level (mg/dl)	7.3 ± 2.4	5.0 ± 2.7	< 0.001*

% within (ROP/no ROP) groups

*significant at *p* value less than 0.05

*ROP* Retinopathy of Prematurity

### Neonatal therapeutic interventions

Twenty-three of 71 infants who received blood transfusion developed ROP, compared to only four of 44 infants who did not receive blood transfusion (*P* = 0.016). The incidence of ROP was not statistically significant among infants who were given surfactant (*P* = 0.65). It was found that giving oxygen supplements is significantly associated with the development of ROP (*P* < 0.0010) either by the non-mechanical “C-pap or nasal cannula” (*P* < 0.0010) or mechanical ventilation methods (*P* = 0.0070). Refer to Table 4.

**Table 4:** Neonatal therapeutic interventions

Variable	No ROP % (*n* = 88)	ROP % (*n* = 27)	*P* value
Blood transfusion	48 (54.5%)	23 (85.2%)	0.016*
Surfactant	34 (38.6%)	13 (48.1%)	0.65
Total days on non-mechanical ventilation	9.5 ± 10.3	20.8 ± 18.1	< 0.0010*
Total days on mechanical ventilation	3.1 ± 10.0	10.8 ± 19.2	0.0070*
Total days on oxygen supplement	12.6 ± 15.9	31.6 ± 23.8	< 0.0010*

% within (ROP/no ROP) groups

*significant at *p* value less than 0.05

Multivariate analysis of statistically significant risk factors is shown in Table 5. Only gestational age (*P* = 0.040, AOR = 0.3, 95% CI = 0.0160–0.956), maximum bilirubin level (*P* = 0.0090, AOR = 0.527, 95% CI = 0.325–0.854) and total days on oxygen supplement (*P* = 0.034, OR = 1.119, 95% CI = 1.008–1.241) were independent predictors for developing ROP.

**Table 5:** Multivariate analysis

Risk factors	AOR	95% CI	*P* value
Gestational age (weeks)	.393	.159–.972	.043*
Birth weight (grams)	.999	.994–1.003	.58
Multiple gestation	1.376	.561–3.372	.49
Sibling affected by ROP	.761	.136–4.261	.76
Level of hemoglobin at birth (g/dl)	1.089	.734–1.614	.67
Respiratory distress syndrome	.862	.176–4.224	.86
Lowest hemoglobin level (g/dl)	.764	.387–1.506	.44
Maximum bilirubin level (mg/dl)	.528	.322–.867	.012*
Blood transfusion	2.642	.660–10.576	.17
Total days on mechanical ventilation	.963	.868–1.069	.48
Total days on non mechanical ventilation	.983	.851–1.134	.81
Total days on oxygen supplement	1.120	1.006–1.246	.038*

*significant at *p* value less than 0.05

*AOR* Adjusted Odds ratio

*CI* Confidence interval

*ROP* Retinopathy of Prematurity

## Discussion

The incidence of ROP and severe type 1 ROP in our study was 23.5 and 11.3% respectively. It is considered a relatively low percentage compared to neighboring countries that have different levels of human development index (Refer to Table 6). The improvements in neonatal care resulted from well recognizing of risk factors and pathogenesis of ROP could be the reason for this relative lower percentage of ROP in Palestine. Conservative use of supplemental oxygen and meticulous monitoring of blood oxygen levels are probably the most important factors that are responsible for the lower risk among Palestinian infants.

**Table 6:** The incidence of ROP in nearby parts of the world

Site of study	Year of publication	Incidence	Level of Human development
Palestine (this study)	2019	23.5%	Medium
Oman [17]	2016	46.4%	Very high
Kuwait [18]	2010	38.9%	Very high
Saudi Arabia [19]	2016	33.7%	Very high
Bahrain [7]	2015	20.4%	Very high
Jordan [20]	2011	28.6%	High
Egypt [21]	2015	36.5%	Medium
Sudan [22]	2014	37%	Low

*ROP* retinopathy of prematurity

The screening guidelines developed in highly developed countries that may differ in term of many aspects such as resources, awareness so these guidelines might not be outfit all situations, the finding that bigger, more mature infants are developing threshold ROP in less developed countries or present too late for treatment [15], and the lack of independent screening criteria in Palestine guided us to widen our inclusion criteria. The criteria suggested screening examinations be performed on children up to and including 32 weeks postmenstrual age (PMA) and infants of less than 1500 g birth weight, or infants with unstable clinical course between 1500 and 2000 g, or any infant that the neonatologist considers at risk because of unstable clinical course.

The mean gestational age for those who developed ROP in our study was 29.4 ± 1.3 weeks. This was a statistically significant difference when compared with the Non-ROP group (*P* < 0.0010) even after multivariate analysis. Compared with other countries with similar human development index, the mean gestational age for those who developed ROP –in weeks- was found to be 30.18 ± 2.28 in Iran [23], 30.00 ± 1.67 in Egypt [21], and 28.9 ± 2 in Jordan [20]. In our study, only 6.3% of infants (4 babies) who had a gestational age more than 30 weeks developed ROP. 2 of them also had a birth weight above 1500, which is above the recommended weight for screening by the recent American Academy of Pediatrics (AAP) and American Association for Pediatric Ophthalmology and Strabismus (AAPOS) guidelines [24]. A total of 2 cases –none of them developed severe type 1 ROP- would have been missed. However those could be guided to screening according to the instability of their clinical course, as both infants were on oxygen supplementation for more than a few days according to the AAP/AAPOS guidelines. Further studies must be conducted to examine the eligibility of such or other guidelines in Palestine. Regarding birth weight, the average –in grams- in those who developed ROP was 1185.0 ± 308.3. While in the non-affected one was 1382.5 ± 253.3 (*P* = 0.0012). This difference was of no significance when controlled to other variables in a multivariate analysis.

Our results were consistent with other studies that suggested that the incidence and severity of ROP are inversely related to birth weight and gestational age, with a few diagnoses of severe ROP (stage 3, threshold disease, or greater) being identified among infants with a birth weight > 1500 g or gestational age > 32 weeks [25]. In population-based studies, the incidence of severe ROP was previously related as being higher among infants of less than 28 weeks’ gestational age or weighing less than 1000 g [26, 27].

Being a singleton or a part of multiple gestation was not found to be of significance regarding the development of ROP in our study (*P* = 0.75). This was also established in many other studies [9, 23, 28]. However, being a part of multiple gestations and having a sibling –from the same gestation- affected by ROP is associated with increased risk for development of ROP in univariate, but not in the multivariate analysis.

The study found that lower average hemoglobin level at birth was found to be associated with the development of ROP and also, among those without ROP, the lowest hemoglobin level before the date of first examination was found to have an average of 8.4 ± 1.4 g/dl, while in the affected group it was 7.8 ± 1.5 g/dl (*P* = 0.038). Both variables were of no significance in multivariate analysis.

The study also found on univariate analysis that blood transfusion was a significant risk factor for ROP development and this was similar to other studies [27, 29]. This can be explained by the fact that blood given to infants contains high level of 2,3-diphosphoglycerate in addition to adult RBCs, which have less affinity to oxygen, thus allowing more delivery of oxygen to retinal tissue [30]. In contrast, a systematic review study found that by adjusting the gestational age and birth weight, the association between blood transfusion and ROP becomes less significant [31]. This was similar to our study as blood transfusion was not significant in multivariate analysis.

Many neonatal conditions and its association with development with ROP were studied (i.e. sepsis, intraventricular hemorrhage, patent ductus arteriosus and hydrocephalus, respiratory distress syndrome, hyperbilirubinemia, use of surfactant). Many studies have suggested their association with ROP development [19, 32], while others did not [33, 34]. Among them, respiratory distress syndrome was the only variable that showed statistically significant association with the development of ROP but only in univariate analysis. This was consistent with Reyes Z’s et al. 2014 study [32].

However, our results showed that the higher the maximum bilirubin level, the less the occurrence of ROP and this relation was consistent even after controlling for other factors. Meanwhile, other studies reported that hyperbilirubinemia has no association with ROP status [35, 36]. *Heyman* et al. [37], who has the same finding, suggested that bilirubin may have a protective role on the development of ROP, due to it acting as a physiological anti-oxidant.

Regarding the duration of oxygen supplement i.e. either by mechanical, non-mechanical or as a total, all were found as significant risk factors for ROP on univariate analysis in our study. However after controlling for other factors in a multivariate analysis, only the duration of total oxygen supplement was still showing significance. Our results had conflicting results compared to previously published studies. Hekeem et al. found that oxygen therapy is a significant risk factor but duration of its use carries no significance [30]. A Brazilian study, on the other hand, showed that giving supplemental oxygen by either headpiece or cpap for more than 5 days was not associated with the development of ROP, while giving oxygen by mechanical ventilation for more than 5 days was of statistical importance. However, by multivariate analysis, even mechanical ventilation method was no longer considered as a risk factor [28].

### Limitations

The study was conducted in a retrospective way; this was the most important limitation. Relevant data were collected from the infant’s medical records, which may possibly contain some errors in documentation. Due to the logistic difficulty of obtaining data from other NICUs in Palestine i.e. Jerusalem and Gaza only three out of 27 NICUs were involved, this may not represent the whole population accurately [38].

## Conclusion

This study represents the first study in Palestine that analyzed epidemiological aspects of ROP. The incidence of ROP is considered a relatively low percentage compared to neighboring countries that have higher levels of human development, and it confirms two risk factors recognized by previous statistical analysis: low gestational age and total days on oxygen supplement. High bilirubin level was suggested to act as a protective factor against the development of ROP. The screening criteria used are suitable to detect all ROP cases effectively. However, more comprehensive studies are needed to set up a national screening guideline for ROP cases.

## Data Availability

These data represent information collected from patient files only. The personal information collected from the files will be secured and based on the ethical approval from An-Najah National University’s IRB ethical committee. The data sets used and/or analyzed during the current study available from the corresponding author on reasonable request.

## Abbreviations

AAP: American Academy of Pediatrics
AAPOS: American Association for Pediatric Ophthalmology and Strabismus
G: Grams
IRB: Institutional Review Board
LMP: last menstrual period
NICUs: Neonatal intensive care units
PMA: Postmenstrual age
ROP: Retinopathy of Prematurity
SPSS: Statistical Package for Social Sciences
